# What Happens to Adults, From Different Socio-Economic Backgrounds, Who Join a Community Weight Management Programme? Long-Term Outcomes up to Nine Years After First Joining

**DOI:** 10.1177/21501319261462639

**Published:** 2026-06-15

**Authors:** Amanda Avery, Josef Toon, Sarah Bennett, Carolyn Pallister, Jemma Donovan

**Affiliations:** 1 Division of Food, Nutrition & Dietetics, School of Biosciences, 6123 University of Nottingham, Nottingham, UK; 2 Nutrition, Research & Health Policy Group, 365306 Slimming World, Derbyshire, UK; 3 School of Applied Social Science, 4487 DeMontford University, Leicester, UK

**Keywords:** long-term weight management, community weight management, obesity, real-world, evaluation

## Abstract

**Introduction:**

Real-world behavioural interventions are required that support long-term weight loss maintenance. This study evaluates the most recent weight and body mass index (BMI) outcomes of adults who first joined a community weight management programme in 2016 and accessed support >two years later.

**Methods:**

Electronic records enabled matching of data for members engaging/re-engaging between 2018 and January 2025.

**Results:**

Of 100,393 adults, (8.5% male), mean age and BMI at baseline were 49.4 (13.79) years and 33.9 (6.82) kg/m^2^. 93.7% were matched to an Index of Multiple Deprivation (IMD) quintile with 15.1% (n=14,238) in the 20% most and 24.3% (n=22,839) in the 20% least deprived. Across all years of last attendance, a minimum mean weight loss of >10% was achieved. Those last attending in 2024 achieved/maintained a weight loss of 13.7 (8.86) % and BMI reduction of 4.85 (3.83) kg/m^2^. Reaching a personal target in 2016, being at target weight at follow-up and the level of target set all predicted greater weight loss maintenance (p<0.001). Socioeconomic status explained little of the overall variance, but members from Q1 (most deprived) achieved greater weight loss maintenance than members from Q5 (least deprived) (p<0.02).

**Conclusion:**

Where extended data was available, this study shows that adults living with excess weight at baseline were able to maintain a clinically beneficial weight loss of >10% for up to nine years after support was first accessed. The importance of setting targets in behavioural weight management interventions is demonstrated. Members from more socially disadvantaged IMD quintiles achieved greater weight losses over the extended follow up suggesting the support was appropriately tailored to meet different needs.

## Introduction

Obesity is a chronic, progressive and relapsing condition and as such, people living with overweight and obesity (PLwO) require on-going or intermittent support over time. Adults, especially females, from disadvantaged backgrounds are more likely to have excess weight associated with poorer physical and mental health.^1^

Well-designed behavioural interventions typically result in clinically significant weight loss outcomes of 5-10% at 6-months for 50-67% of adults who participate in the trials.^2^ However, the increasingly obesogenic environment and the individual pre-disposition towards adiposity, combined with the normal physiological response to reduced energy intake, make weight loss difficult to maintain.^3^ Lost weight can be easily regained, and studies suggest only 15-25% of individuals achieve and maintain a 10% or greater weight loss.^3^ The responses to any non-surgical or surgical interventions are highly heterogeneous, but most follow a typical pattern of an initial weight loss period of around 6–9 months followed by a variable period of relative weight stability and then weight regain.^4^ Studies consistently report the need for behavioural changes (increased exercise and sustained dietary changes) for successful long-term maintenance of reduced body weight with minimum weight regain. Qualitative research suggests that sustained and substantial weight loss is facilitated by continuous self-monitoring and structured goal setting, underpinned by persistent motivation and positive reinforcement, while effectively managing ongoing challenges and mitigating the impact of discouraging experiences.^5^

Several research trials, typically including interventions supporting intensive lifestyle change, to encourage healthier behavioural habits, have been undertaken to investigate how weight loss maintenance (WLM) can best be supported.^6-8^ However, the follow-up period does not usually extend for longer than two years. Exceptions include the WRAP trial where patients from primary care were randomised to receive a brief intervention or support from a commercial behaviour change based weight management programme (WW, Weight Watchers) for either 12 or 52 weeks. At five-year follow-up, those adults randomised to the WW intervention were around 2kg lighter, 1.95kg lighter for the 12-week and 2.67kg lighter for the 52-week intervention, compared to 0.46kg lighter for the brief intervention group.^9^

Previous meta-analyses have suggested that people participating in WLM interventions experience less weight regain compared to controls but there is limited evidence on the long-term sustainability of these interventions.^10^ Furthermore, it is unknown whether extending the duration of an effective WLM intervention results in better outcomes. The US Weight Loss Maintenance trial found slightly greater weight loss outcomes at five years in the adults receiving personal contact support compared to those assigned to the self-directed group but no additional benefit if the support was extended after 30 months.^11^ Scalable WLM interventions for PLwO are lacking but vital for the health and economic benefits of weight loss to be fully realised. The NULevel trial investigated the effectiveness of a low-intensity technology-mediated behavioural intervention to support WLM in adults after clinically significant weight loss (≥5%) compared to standard lifestyle advice. After 12-months there was no difference in the WLM of the participants who received the intervention compared to standard lifestyle advice via a newsletter.^7^

Many current real-world interventions only offer support for a relatively short period of time with very limited support once the individual has come to the end of their commissioned support and with very limited follow-up evaluation. Commercial programmes can reach a large proportion of the UK population who may benefit from both weight loss and WLM and are clinically recommended.^12^ Furthermore, they can offer participant flexibility in adherence and treatment duration allowing on-going or intermittent support according to the PLwO’s needs given the chronic and relapsing nature of obesity. Some programmes offer incentives to members who achieve and maintain a personal target weight, enabling continued access to the support to help prevent weight regain.

However, longer-term follow up outcomes of people receiving support from such programmes in self-funded real-world settings are understudied. This study evaluates the most recent weight outcomes of adults who first joined a community weight management programme, in 2016 and accessed support from the programme at least two years later to address the following research questions:

1) What weight and BMI changes have been achieved and maintained by adults up to nine years after first joining a community weight management programme in 2016
2) How do weight loss targets influence weight changes up to nine years after joining a community weight management programme
3) How do age, gender, BMI at baseline and socioeconomic status influence long-term weight change

## Methods

### Study Population

The dataset used for this study was of self-funding, non-pregnant adults, (aged 18-80 years), from the United Kingdom and the Republic of Ireland, who joined the community weight management programme during 2016 and who had at least one attendance weight entered when they first joined. Analysis of weight change over the course of one year’s attendance for this dataset has previously been reported.^13^

This secondary analysis examined the most recent weight data for adults with continuing records with the programme at least two years after first joining in 2016 i.e. who then attended between 2018 and 2025. The year of last attendance and associated weight data for members attending between 2018 and January 2025 were extracted. Of note, the most recent weight recorded does not look comprehensively at an individual’s weight journey. Individuals have not necessarily attended non-stop during the follow-up period but may have re-joined to access further support as and when needed.

### Intervention

The community group-based weight management programme consists of a multi-component approach, catering for individual and family needs. It encourages members to eat a healthy diet, cook meals from scratch and make gradual increases in physical activity. The programme uses evidenced-based behaviour change techniques. Members receive support from trained group facilitators and have access to a variety of tools to help them develop and maintain long-term healthier lifestyle changes to maintain weight loss. For example self-regulation strategies are encouraged through weekly recording of weight, the option to use food and activity diaries to monitor and reflect on food intake and physical activity, and individual motivation and peer support to help participants become more confident in changing their mind-set and to develop action plans and goal setting (https://www.slimmingworld.co.uk/how-it-works). Trained group facilitators provide concomitant support through a social environment that avoids criticism, prescriptive control and judgement and instead focuses on facilitating self-guided support to maximise individual member’s goals. Additional online support is available to complement the weekly group-based support. At the time of joining (2016) there were 19,000+ weekly groups, across the United Kingdom and Republic of Ireland, supporting more than 1 million adults, (and indirectly their families), each week.

### Outcome Measurements

Calibrated scales with a precision of ± 0.23 kg (SECA bespoke model) are used within group to record weight and this data is securely and electronically stored within the community weight management provider’s database. Weight is recorded at baseline when the member enrols and at each subsequent attendance. Height is self-reported at baseline and used to calculate BMI using the formula weight (kg)/height (m).^2^

Not all members will want to set a target weight despite being encouraged to think about what weight they would like to be. Where set, target weights are electronically recorded and updated dependent on individual preference with some members preferring to set a series of smaller target weights and other members preferring to just set the one ‘larger’ target. If a member is successful in achieving and maintaining their personal target weight, they can access continued support at no cost for as long as they remain within ±3lbs of their target weight.

Where postcode data were available (excluding Republic of Ireland), socioeconomic status was measured by matching records to an Index of Multiple Deprivation (IMD) quintile.^14^ An IMD quintile ranks areas from 1 to 5, with 1 indicating a postcode area that is located within the 20% most deprived areas and 5 indicating a postcode area is located within the 20% least deprived areas.

### Statistical Analysis

All data were analysed using R and described using descriptive and inferential statistics. Multiple regression was used to model weight change at follow-up by regressing the demographic baseline variables e.g., age and BMI (both mean-centred given data range), and IMD quintile and gender and weight data (percentage target weight change, follow-up year, binary data of whether a participant achieved a target weight within the first year of joining the programme and whether they were at target at follow-up). Target weight change was also mean-centred in the analysis.

Given the data does not reflect continuous follow-up as the community weight management programme allows for individuals to leave and rejoin at any time, we could not determine the actual length of time each participant attended the programme and therefore the data were not suitable for survival analysis. Instead, as the study was focused on understanding the weight and BMI changes of those who were attending the programme, multiple linear regression was used to model the impact of demographic and socio-economic variables in addition to the impact of targets with year of last attendance entered to try and unpick the outcomes of those with more recent versus distant last attendances.

### Ethical Considerations

This retrospective, longitudinal study examined long-term weight outcomes in adults first joining the community weight management programme during 2016. This analysis was categorised as a service evaluation, designed to answer the question “What standard does this service achieve,” in accordance with the Health Research Authority’s^15^ definitions of service evaluation, clinical audits and usual practice. Ethical approval was not required given that the primary research was a service evaluation with no new intervention being tested. Upon joining the Slimming World programme, all members are asked for consent for their anonymised data to be used for research and service evaluation purposes. The weight management provider have an internal governance framework and privacy promise, which aligns with the guidelines outlined in the Declaration of Helsinki, that explains to members:

• What we use their data for and why
• How data is processed
• Who processes the data
• Who we share data with and why
• What rights members have/how to exercise them
• What we do to protect their personal information

**Figure 1.**
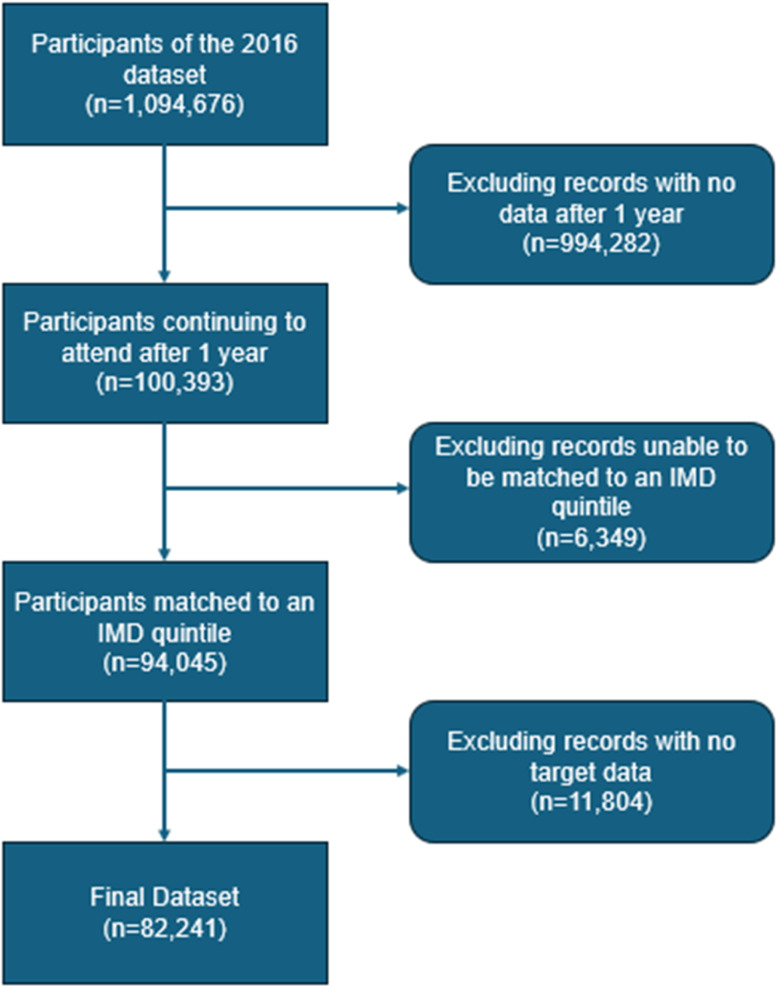
Adapted STROBE reporting diagram showing the inclusion process

STROBE reporting guidelines for cohort studies were adhered to.^16^ An adapted STROBE reporting diagram detailing the inclusion process can be seen in Figure 1.

## Results

Members who had at least one weight recorded between January 2018 and January 2025 (100,393 adults, 9.2% of people who joined in 2016 where their data could be matched), were included in this secondary analysis. Mean age and BMI at baseline were 49.4 (*SD*=13.79) years and 33.9 (*SD*=6.82) kg/m^2^ respectively and 8.5% were male. 93.7% were matched to an IMD quintile with 15.1% (n=14,238) in the 20% most deprived and 24.3% (n=22,839) in the 20% least deprived quintile at baseline.

Data on personal target weights were available for 81.9% (n=82,241) of the dataset. The mean desired target weight equated to a weight change of -20.1% (*SD*=8.72). At the time of their last recorded weight, 20.2% (n=17806) were at their personal target. Those at a their target weight were older (*M*=53.6 years, *SD*=13.62, *Mdn*=55.0, *z*=45.0, *p*<.001) and had a lower baseline BMI (*M*=30.1kg/m^2^, *SD*=4.84, *Mdn*=29.1 kg/m^2^, z=-78.51, *p*<.001) than those not at a personal target weight (*M*=48.4 years, *SD*=13.68, *Mdn*=49.0, and *M*=33.9 kg/m^2^, *SD*=6.47, *Mdn*=32.8 kg/m^2^). Mean change in weight and BMI at follow-up was -11.2% (*SD*=7.94) and -3.9 kg/m^2^ (*SD*=3.24).

Table 1 shows descriptives for changes in weight and BMI at follow-up.

**Table 1.** Descriptives for Mean (M) Changes in Weight (%) and BMI (kg/m^2^) at Follow-Up

​		N	%	Weight change (%)		BMI change	
				*M*	*SD*	*M*	*SD*
Total		100393	​	-11.16	7.94	-3.91	3.24
Gender	Male	8516	91.52	-11.08	8.06	-4.15	3.49
	Female	91877	4.48	-11.16	7.92	-3.89	3.22
Target at 12 months	Yes	45311	54.87	-11.74	7.38	-3.87	2.94
	No	55082	45.13	-10.68	8.34	-3.95	3.48
Target at follow-up	Yes	18072	20.41	-15.68	7.88	-4.98	3.35
	No	70467	79.59	-10.44	7.65	-3.71	3.16
IMD quintile at baseline	Q1	14238	15.14	-11.73	8.58	-4.33	3.64
	Q2	17563	18.68	-11.34	8.13	-4.07	3.40
	Q3	18957	20.16	-11.32	8.01	-3.97	3.27
	Q4	20448	21.74	-10.97	7.71	-3.77	3.06
	Q5	22839	24.29	-10.79	7.51	-3.66	2.97
Last attendance year	2018	50324	50.13	-10.87	7.51	-3.8	3.02
	2019	20717	20.64	-11.2	7.94	-3.93	3.27
	2020	14267	14.21	-10.97	8.3	-3.88	3.41
	2021	5269	5.25	-11.43	8.85	-4.04	3.66
	2022	2506	2.50	-11.12	8.34	-3.86	3.32
	2023	1371	1.37	-11.14	8.6	-3.95	3.53
	2024	5939	5.92	-13.65	8.86	-4.85	3.83

**Figure 2.**
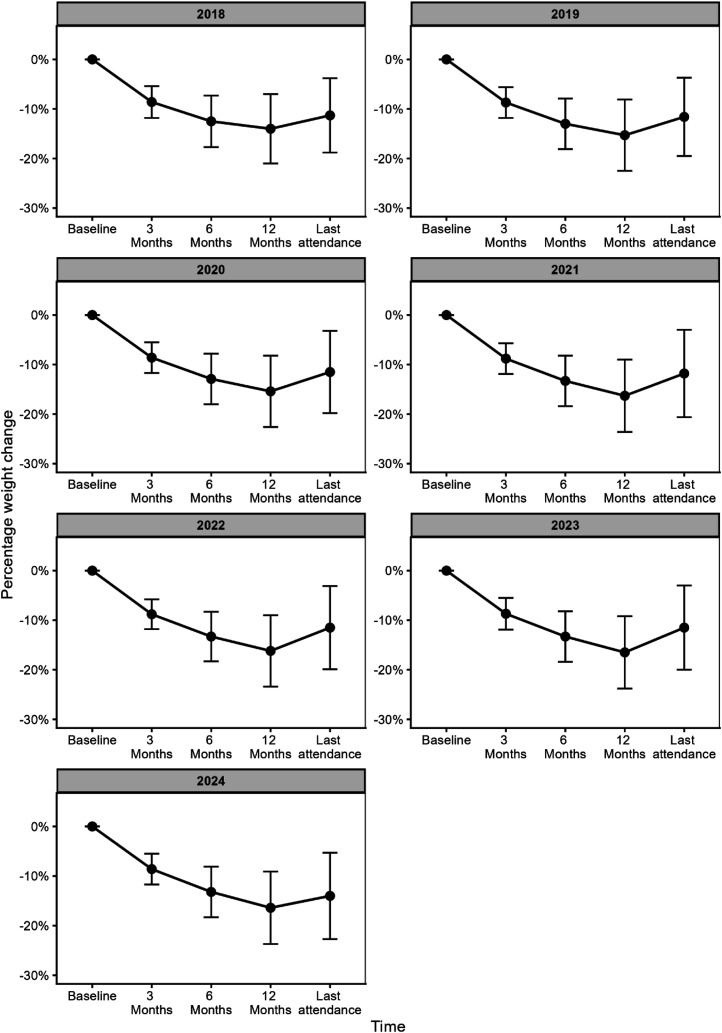
Mean weight change (%) at baseline, 3, 6, 12 months after first joining in 2016, and at follow-up split by year of last/on-going attendance. *Note.* Error bars represent standard deviation (SD)

Figure 2 illustrates the mean percentage weight change at 3, 6 and 12 months after first joining in 2016 and at the most recent attendance.

Multiple regression examined the predictors of percentage weight change amongst the demographic and weight data. Baseline age, BMI and target weight loss were mean centred. IMD quintile at baseline was entered as a categorical variable with Q1 as the reference level. The regression model was significant (*R*^2^=.46, *F*(11, 82229)= 6206, *p*<.001), explaining 46% of the variance in weight loss at follow-up. Model coefficients and percentage variance explained by the predictors are shown in Table 2. The analysis showed that for each year older than the mean of 49.4 years, was associated with a greater weight loss of 0.03% and a target weight loss 1% above the mean of 20.1% was associated with a greater weight loss of 0.63%. Gender did not result in a significant difference but having a baseline BMI of one unit greater than the mean of 33.9 kg/2 was associated with a lesser weight reduction of 0.07%. Whilst baseline IMD quintile data explained very little of the overall variance, members from Q1 (20% most deprived) accessing follow-up support achieved significantly greater weight losses than members from Q5 (20% least deprived) (p<0.02).

**Table 2.** Multiple regression predicting percentage weight change using last observation carried forward (LOCF) data

​	β	SE	95% CI	t	p	% Variance explained
Intercept	-8.82	0.07	[-8.95, -8.68]	-130.97	<.000*	-
Baseline age^	-0.03	0.00	[-0.03, -0.02]	-18.85	<.000*	0.05
Baseline BMI^	0.07	0.00	[0.06, 0.08]	13.59	<.000*	9.27
Gender (Male)	-0.07	0.07	[-0.21, 0.08]	-0.90	.370	0.15
Reached target in 2016 (Yes)	-2.37	0.05	[-2.46, -2.28]	-52.67	<.000*	2.91
Target weight loss^	-0.63	0.00	[-0.63, - 0.64]	-181.18	<.000*	21.35
Target at follow-up (Yes)	-7.09	0.05	[-7.21, -6.99]	-131.52	<.000*	11.62
Last attendance year	-0.02	0.01	[-0.05, -0.01]	-2.11	.035*	<0.001
IMD: Q2	-0.01	0.07	[-0.15, 0.13]	-0.19	.847	0.01
IMD: Q3	-0.08	0.07	[-0.22, 0.06]	-1.16	.248	
IMD: Q4	0.07	0.07	[-0.07, 0.20]	0.99	.324	
IMD: Q5	0.16	0.07	[0.03, 0.29]	2.33	.020*	

Final model n = 82,241, ^mean-centred variable.

*statistically significant.

## Discussion

This real-world evaluation shows successful long-term weight outcomes, in a mainly female adult population living with obesity (BMI ≥30kg/m^2^), from diverse socioeconomic backgrounds who joined the community weight management programme in 2016 and where follow-up data was available two to nine years after joining (between 2018 and 2025). Results show that these members achieved and/or maintained weight losses of over 10% with around 20% attending at their personal target weight at their last recorded weight. This level of weight loss represented a mean reduction of over 4.0 BMI units irrespective of the length of extended follow-up.

From the regression analysis we can see that the level of target weight loss and being at a personal target weight at the time of follow-up, explained most of the variance in weight change, with a third of the total variance explained by these two variables. Achieving a personal weight target within the first year of the programme predicted greater maintained weight loss as did setting a higher desired target weight loss. Of note was that the target weight loss was mean centred in the regression model with the mean personal target weight loss being over 20%. These findings would suggest that having an ambitious target weight can be an important behavioural change strategy to support a greater weight loss that is maintained long-term and some people may benefit from setting an aspirational target of >20%.

Our findings concur with the acknowledgement that the setting of targets or goals is motivating, energising and an important behavioural change technique in weight management interventions.^17^ Weight‐loss targets are important because they regulate behaviour by affecting attention, decisions, effort and task persistence.^18^ They energise and direct behaviour^19^ and create a framework through which a behaviour is perceived and evaluated.^20^ It is routine clinical practice, based on current guidelines, to encourage individuals to set a ‘realistic’ weight‐loss target of 5–10% of initial body weight given this level of weight loss is associated with clinically significant health benefits.^21^ Evidence suggests that effective diet and exercise interventions can result in a weight loss of 5–10% of initial body weight over a 12–24-week period^22^ and yet this level of weight loss may be uninspiring to many individuals living with obesity.^23^ Individuals living with excess weight will often enter a weight‐loss programme with an idea of the amount of weight that they would like to lose as shown in this study with 87% of members having set a personal target. Targets are often much higher than what is actually achieved,^24^ which has led to high targets being considered ‘unrealistic’^25^ with concerns that subsequent failure will lead to disappointment, dissatisfaction, decreased effort and relapse. In the current study, the mean personal weight loss target of 20% could generally be considered ‘unrealistic’ within the context of behavioural weight management. However, our data showed the contrary insofar that having a target weight loss above this average significantly predicted greater weight loss at follow-up. These findings support a previous meta‐analysis that concluded that there was no empirical evidence to suggest that setting realistic targets led to greater weight loss, or that ‘unrealistic’ targets had any negative impact on weight^22^ and other real-world studies suggest the same.^26,27^

Age at baseline was associated with a greater weight loss at follow-up. Age was also mean centred in the regression model with the mean age being 49 years. Cross-sectional data from both the National Health and Nutrition Examination Survey in the USA and the Health Survey for England show that whilst obesity is becoming more prevalent across all age groups, there is a trend for greater rises among older (≥55 years) than younger adults, with levels reaching 40% (vs 35%) in the USA^28^ and 36% (vs 24%) in England in 2022.^29^ For older people, having excess weight comes with additional health risks. More than 80% of older people have at least one chronic health problem, and 50% have two or more.^30^ Obesity exacerbates age-related decline in physical function with an excess body fat mass related to increased decline in physical dysfunction and disability.^31^ Thus, maintaining a healthy weight as one approaches 60 years or older is equally important providing the behavioural changes encourage a nutritionally adequate diet and sufficient physical activity to maintain muscle strength and bone health. A qualitative study of adults aged 65+ living in London, where the participants had recent experience of weight management, found that the participants recognised that excess weight has a negative effect on health and well-being. Health and appearance were both considered to be powerful motivators of weight management in this group of older UK adults.^32^

The reported associations between age and weight loss also concur with previous findings where being older predicted greater weight loss in over 1.3 million adults accessing Slimming World weight management support.^33^

Data from this study showed the effect of gender was non-significant and did not impact weight outcomes at follow-up. It has been suggested that obesity manifests differently in men and women^1^ but our findings suggest equally successful weight losses in those male and female members accessing the extended support. We have previously found within this population group, males showed significantly greater weight loss at 3 months but no significant difference in outcomes at 12-months follow-up.^13^ The non-effect of gender within the current study could be explained by the proportion of males and females within the study sample with less than 10% of the study population being male. Whilst low, this proportion is reflective of the gender distribution in membership of the programme at any point in time and is similar to what has been observed within weight management trials.^34^

BMI at baseline explained almost 10% of the variance in weight loss seen at follow-up. A greater BMI at baseline was associated with less weight loss at follow-up. BMI was also mean centred in the regression model with the mean BMI approaching 34kg/m^2^ and thus the clinical interpretation is that a member with a baseline BMI of 40kg/m^2^ would be expected to achieve 0.42% less weight loss at follow-up. This finding is not surprising given the increasing complexity associated with severe obesity.^35^

Socioeconomic status, as reported using baseline IMD quintiles explained very little of the overall variance. However, there was a significant difference in weight change between quintile 1 and 5, showing greater weight loss at follow-up for members living within the 20% most deprived areas compared to the 20% least deprived. With the socioeconomic inequalities surrounding the prevalence of obesity and excess weight^36^ there has been a call for targeted interventions to support this group. Qualitative findings from a study exploring the views of a US inner city population found ‘accountability’ to be important in weight management support.^37^ In Northwest England, an investigation into how best to support people from disadvantaged backgrounds recommended tailoring existing interventions and to include behavioural change techniques such as target setting.^38^ Tailored interventions need to be mindful of cost, cultural diversity and literacy levels. The Slimming World programme is tailored to meet the needs of the local community and both the facilitation and peer support enable members to be able to access an affordable heathier diet with resources available for people with different levels of literacy. The group support, whilst facilitating accountability, has also been shown to enhance self-esteem and levels of mental well-being.^39^ The weight loss observed across IMD quintile within the current study highlights the importance of extended open-ended support as a key factor in successful obesity management in supporting people across all socioeconomic groups.

As with all real-world data, there are some limitations. First, the current study examines the most recently available data for a subset of members who initially joined in 2016 and does not quantify the level of support accessed and whether this was accessed continuously or intermittently. The rationale for extracting the most recent data point was due to challenges associated with reporting within a large-scale open-ended programme where individuals may leave and re-join the programme under a new identifier making connecting historical records difficult. Therefore, it is unlikely that the data presented fully reflects the outcomes of all members who attended during the nine year extended follow up period. It should be noted however that the percentage of people with data available two years after first joining is very similar to the percentage of people followed up in a real-world study of adults using pharmacotherapy as part of a weight management intervention.^40^ Some of the variables, for example IMD quintile reflecting socioeconomic status, used in the regression analysis may have changed over the nine year period.

A further limitation was that the study could not access outcomes in people who may have joined in 2016 and who were either able to continue managing their weight without requiring any extended support, those who proceeded to access other forms of support or those who may still be struggling with their weight after stopping attending the programme. The data collection period was also impacted by COVID and this is clearly reflected by the significant reduction in numbers between 2020 and 2022. When ‘shut down’ was announced in March 2020, in-person groups had to close and transition to online groups. Not everyone found the support offered online to be the same as the in-person group support and camaraderie.^41^ Many people struggled with weight management during COVID and especially people with lower incomes whose ability to maintain healthier behaviours may have been even more affected.^42^ Nonetheless, being able to follow people up for up to nine years in a real-world setting is a strength of this study and there are no other real-world studies, investigating the efficacy of behavioural weight management interventions, that have been able to achieve this. This study suggests that behavioural weight management interventions can support weight loss maintenance and may provide an option for people following cessation of weight loss medications to reduce the extent of weight regain that is commonly seen.^43^

## Conclusion

For those members for whom extended data was available, this study shows that adults living with excess weight at baseline were able to maintain a clinically beneficial mean weight loss of over 10% for a period of up to nine years. Setting a target and being at a personal target weight were significant drivers for successful weight loss, emphasising the importance of setting weight targets in behavioural weight management interventions. Around a fifth of the study population were at a personal target weight. Men and women were equally successful in achieving significant weight loss and those older, more so. Of note, is the fact that those members from the more socially disadvantaged IMD quintiles were able to achieve greater weight losses over the extended period of follow up suggesting that the community-based weight management support was appropriately tailored to meet different needs. The study highlights the importance of extended open-ended group support as a key factor in successful weight management, for people from a range of backgrounds, given the chronic and progressive nature of obesity.

## Data Availability

Data will be made available upon reasonable request to the corresponding author.*
